# Supplementary material for: Exploring the Structural Rearrangements of the Human Insulin-Degrading Enzyme through Molecular Dynamics Simulations
Source: Int J Mol Sci. 2022 Feb 3;23(3):1746. doi: 10.3390/ijms23031746 (PMC8836115; doi:10.3390/ijms23031746)
Supplement: Supplementary file 1 [file ijms-23-01746-s001.zip › ijms-1556575-supplementary.pdf]

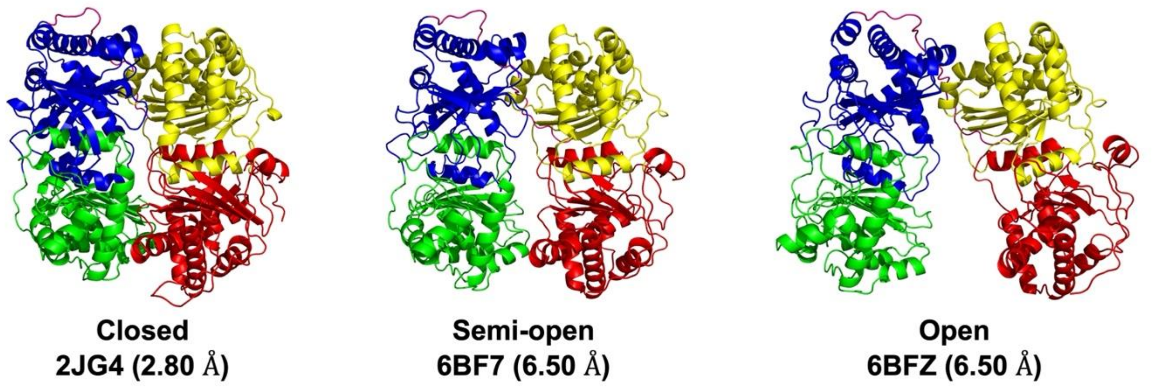

Figure S1. IDE different states that are available in the PDB database.

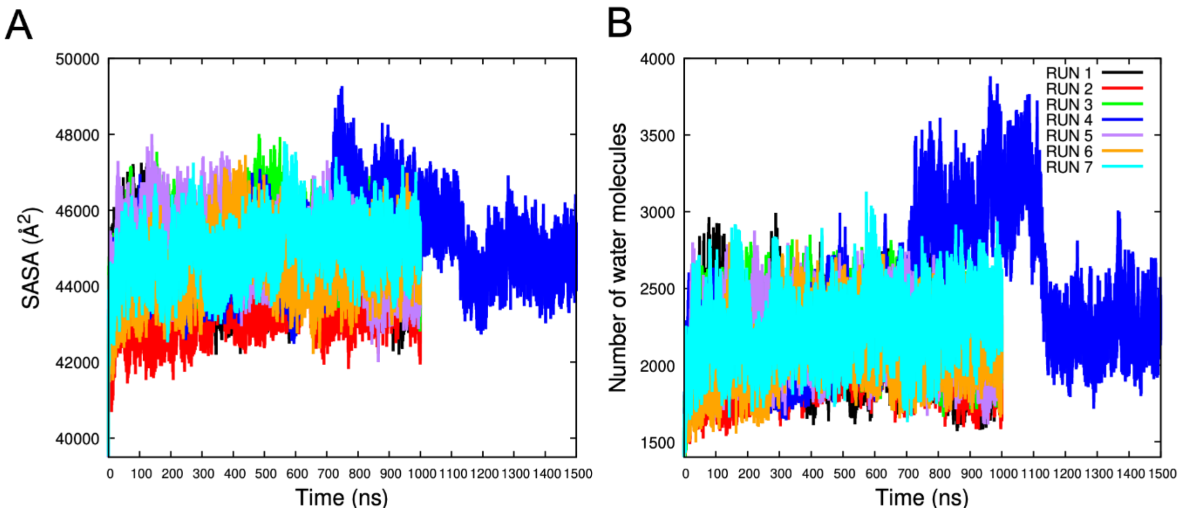

Figure S2. (A) The SASA fluctuation computed during each MD simulations alongside **(B)** The number of water molecules in the cavity of IDE.

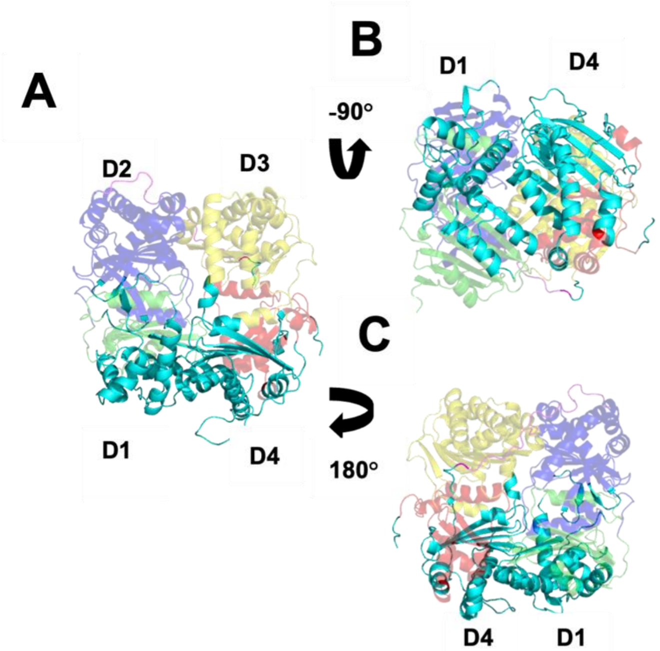

Figure S3. Illustration of the flexible regions of IDE detected after the Cα RMSF analysis. The most flexible regions are represented in cyan. Domain 1 is displayed in green, domain 2 in blue, domain 3 in yellow and domain 4 in red. **(A)** Representation of IDE front side. **(B)** Representation of IDE bottom structure (view side on D1 and D4). **(C)** Representation of IDE back side.

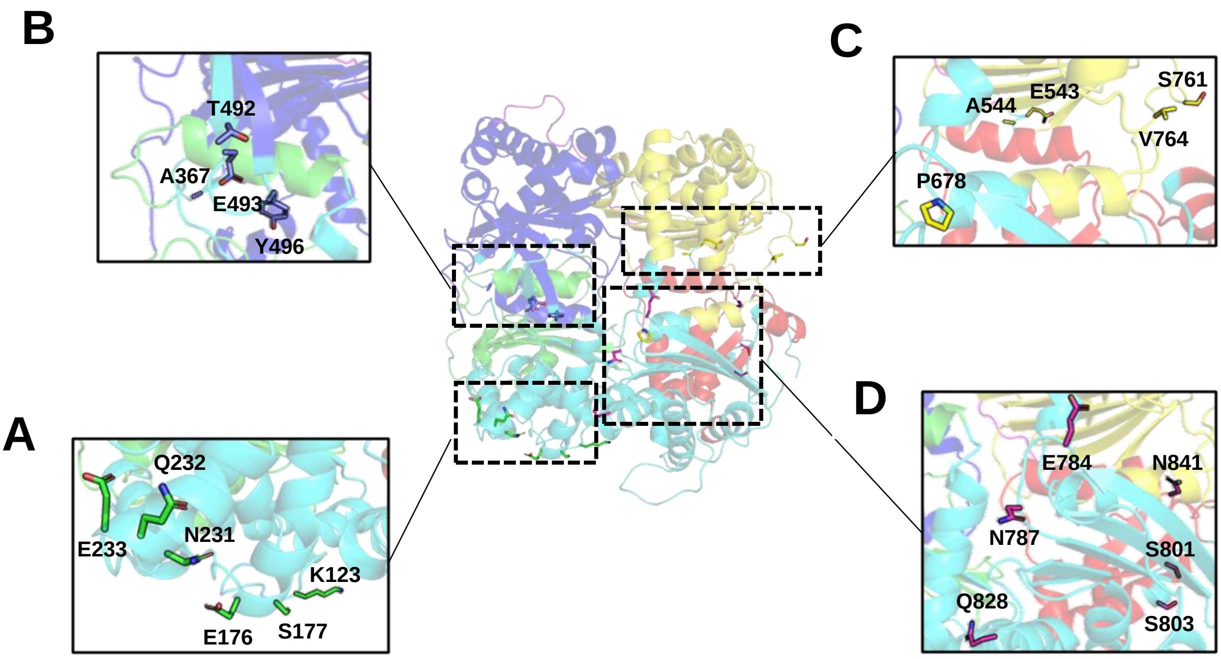

Figure S4. Illustration of the most flexible residues of IDE detected after the Cα RMSF analysis. The most flexible regions are represented in cyan. Domain 1 is displayed in green, domain 2 in blue, domain in yellow and domain 4 in red. All residues are displayed according to the domain color code. **(A)** Representation of D1 residues. **(B)** Representation of D2 residues. **(C)** Representation of D3 residues. **(D)** Representation of D4 residues.

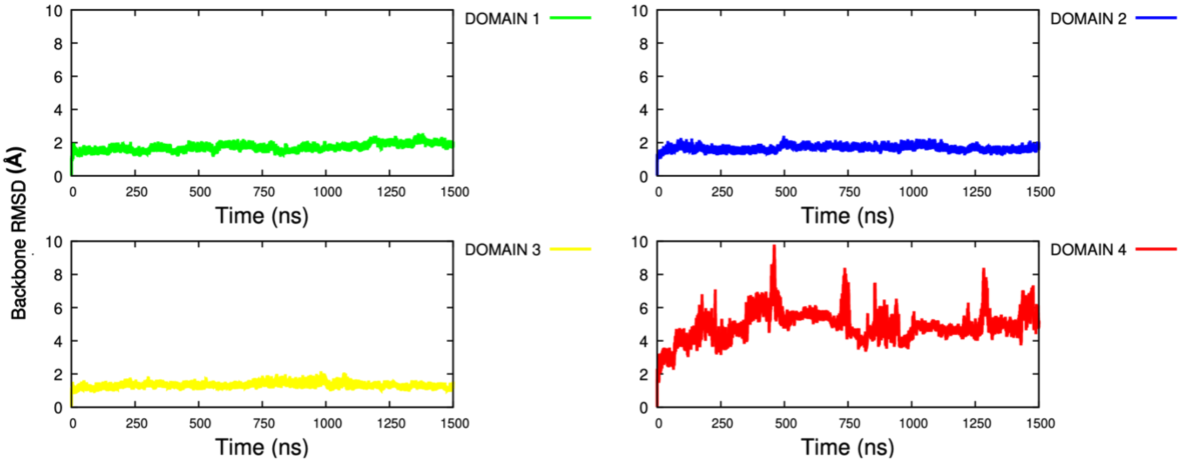

Figure S5. Evaluation of the backbone RMSD of IDE different domains during the fourth trajectory. D1, D2 and D3 display RMSD values ranging between 1 and 2 Å. D4 exhibits higher fluctuations due to its very flexible loops (residues 971-978 and 1012-1019).

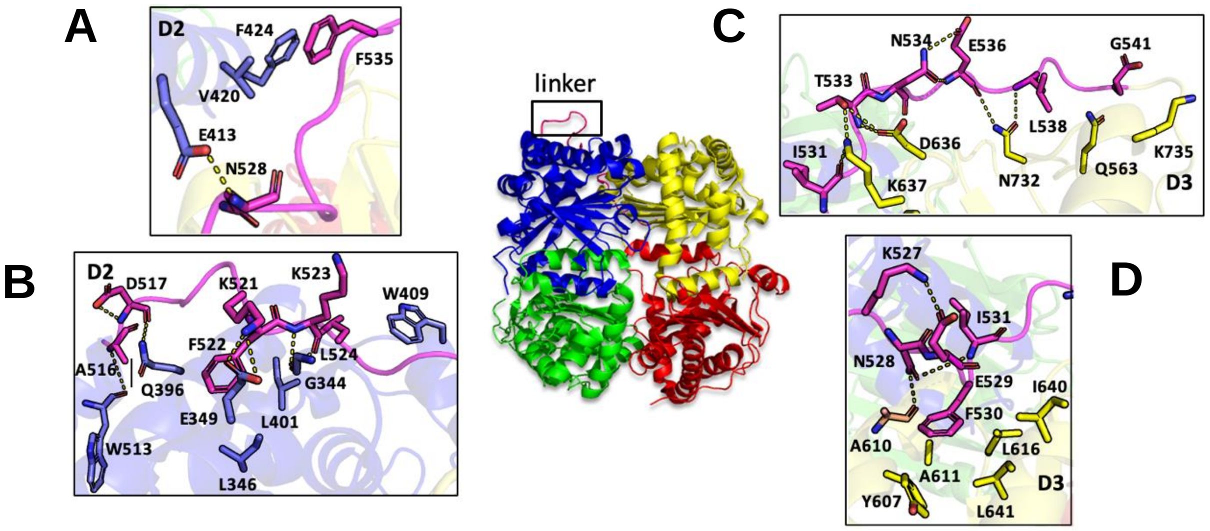

Figure S6. Interaction network of the linker with D2 and D3 of IDE. The color code has been maintained the same for the residues of the different domains. The linker is represented in magenta and all residues are displayed in sticks (most of the residues have only their side chains represented). **(A)** and **(B)** represent the interactions formed between D2 and the linker. As for **(C)** and **(D)**, they represent the interactions made with the loop and D3.

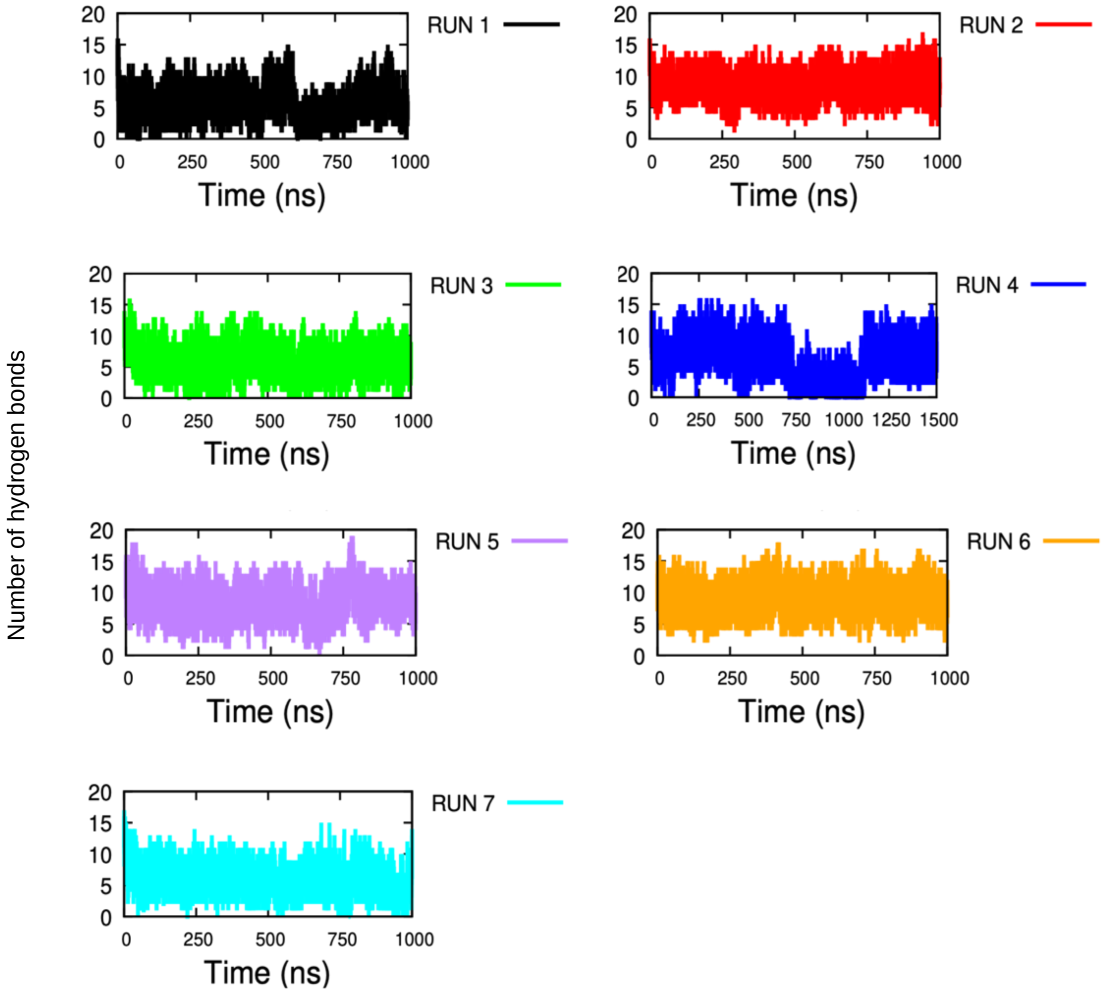

Figure S7. Number of hydrogen bonds formed between D2 and D3 along the MD simulations.

Table S1. D2-D3 non-covalent interactions occupancy (%) during MD simulations. HB and SB were reported only if they exist for >= 10% of the investigated period. Backbone (bb) and side chain (sd) interactions were specified.

Domain 2	Domain 3	Non-covalent interaction type	Occupancy (%)						
			Run 1	Run 2	Run 3	Run 4	Run 5	Run 6	Run7
D309 (bb)	N672 (sd)	HB	0.0	90.2	0.0	18.9	76.1	11.0	0.0
D309 (sd)	N671 (sd)	HB	11.9	11.4	0.0	0.0	0.0	0.0	0.0
D309 (sd)	R668 (sd)	SB	0.0	0.0	0.0	69.8	24.4	0.0	0.0
E382 (sd)	K657 (sd)	SB	37.1	63.4	48.3	35.3	0.0	100	38.9
E381 (sd)	K657 (sd)	SB	32.5	0.0	0.0	0.0	96.7	0.0	0.0
D426 (sd)	K571 (sd)	SB	99.0	65.6	91.8	76.9	86.5	99.7	91.5
F424 (bb)	K571 (sd)	HB	39.0	38.5	48.4	34.1	40.9	42.1	46.1
K351 (sd)	E606 (sd)	SB	21.2	29.5	30.8	0.0	0.0	0.0	31.4
K351 (sd)	D602 (sd)	SB	11.0	100.0	0.0	26.0	73.3	100.0	0.0
H336 (sd)	Y609 (sd)	HB	27.9	0.0	34.4	22.3	27	19.5	25.4
H340 (bb)	Y609 (sd)	HB	0.0	22.7	0.0	0.0	0.0	0.0	0.0
R311 (sd)	E664 (sd)	SB	92.6	72.8	100	81.2	0.0	0.0	100.0

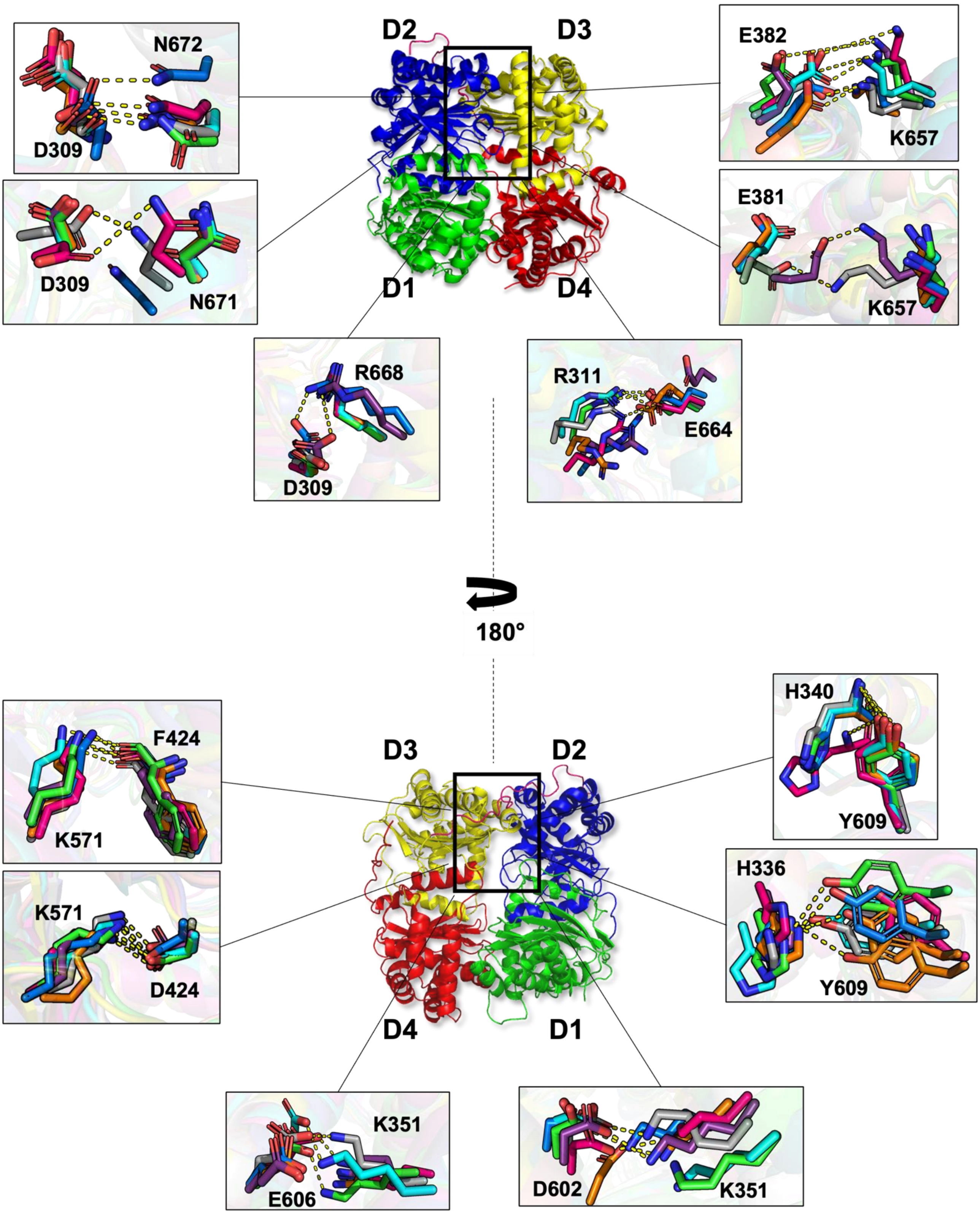

Figure S8. Hydrogen bonds and salt bridges formed between D2 and D3. The color code has been conserved according to the simulation number. Grey is for run 1, red is for run 2, green is for run 3, blue is for run 4, purple is for run 5, orange is for run 6 and cyan is for run 7.

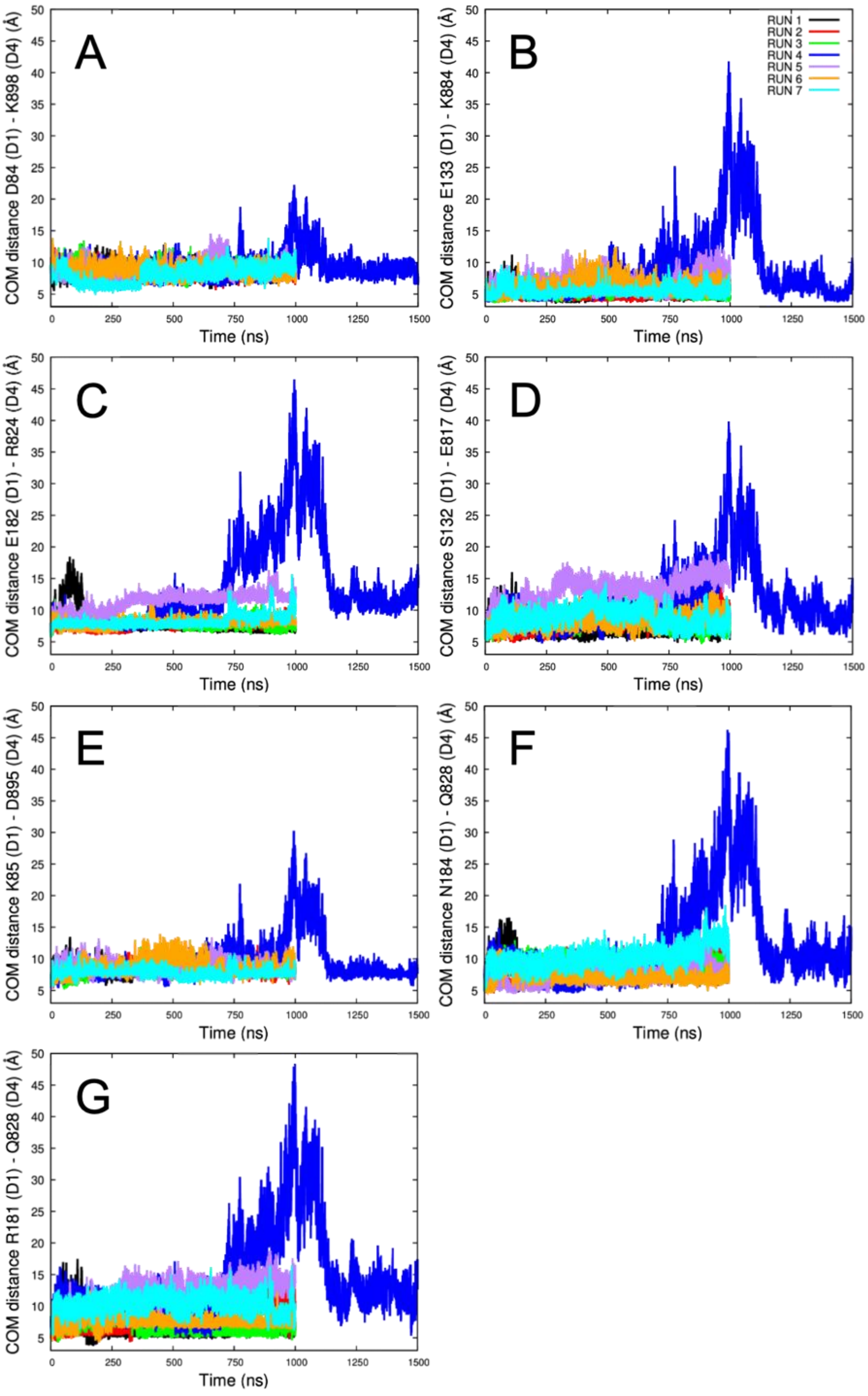

Figure S9. The COM distance over time between key residues of IDE domains 1 and 4 involved in the closed-open switch. **(A)** The COM distance of D84-K898. **(B)** The COM distance of E133-K884. **(C)** The COM distance of E182-R824. **(D)** The COM distance of S132-K898. **(E)** The COM distance of K85-D895. **(F)** The COM distance of N184-Q828. **(G)** The COM distance of R181-Q828.

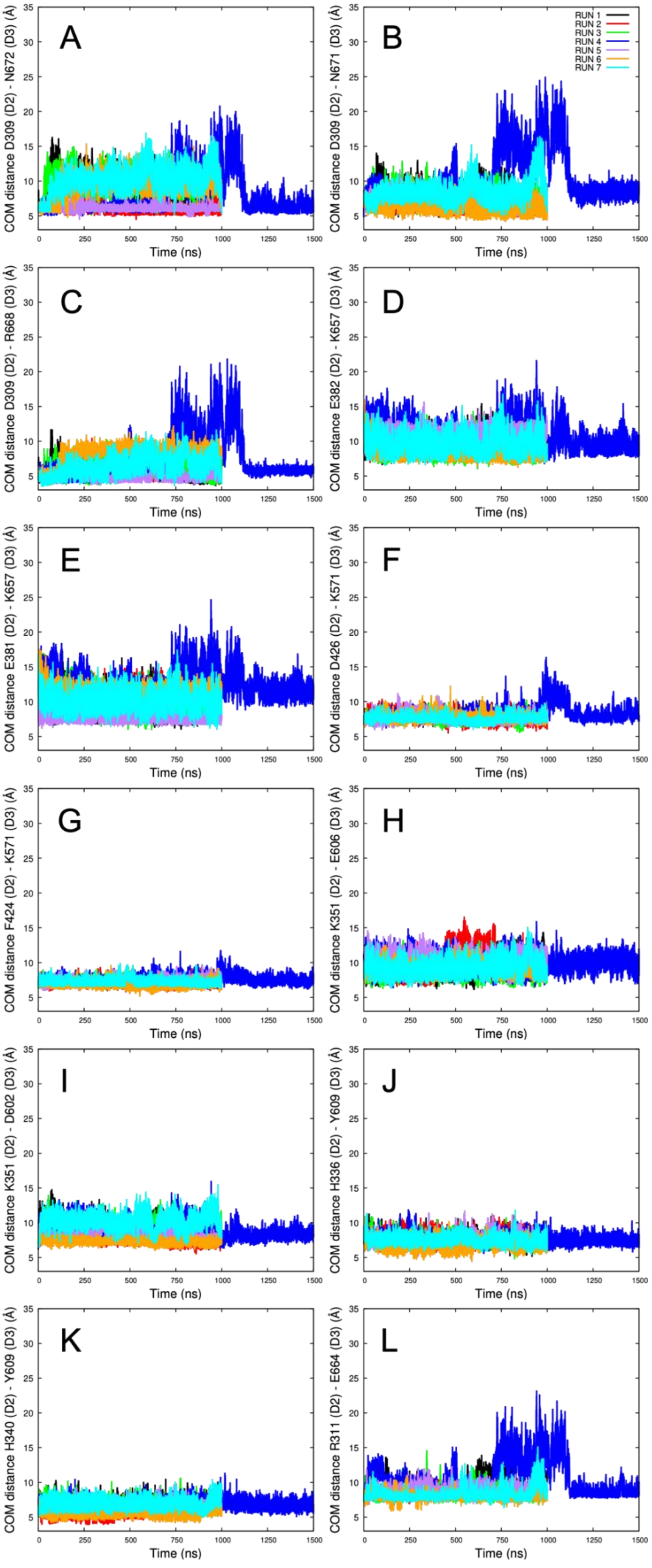
A
B
C
D
E
F
G
H
I
J
K
L
RUN 1
RUN 2
RUN 3
RUN 4
RUN 5
RUN 6
RUN 7
COM distance D309 (D2) - N672 (D3) (Å)
COM distance D309 (D2) - N671 (D3) (Å)
COM distance D309 (D2) - R668 (D3) (Å)
COM distance E382 (D2) - K657 (D3) (Å)
COM distance E381 (D2) - K657 (D3) (Å)
COM distance D426 (D2) - K571 (D3) (Å)
COM distance F424 (D2) - K571 (D3) (Å)
COM distance K351 (D2) - E606 (D3) (Å)
COM distance K351 (D2) - D602 (D3) (Å)
COM distance H336 (D2) - Y609 (D3) (Å)
COM distance H340 (D2) - Y609 (D3) (Å)
COM distance R311 (D2) - E664 (D3) (Å)
Time (ns)
0
250
500
750
1000
1250
1500
5
10
15
20
25
30
35

Figure S10. The COM distance over time between key residues of IDE domains 2 and 3 involved in the closed-open switch. **(A)** The COM distance of D309-N672. **(B)** The COM distance of D309-N671. **(C)** The COM distance of D309-R668. **(D)** The COM distance of E382-K657. **(E)** The COM distance of E381-K657. **(F)** The COM distance of D426-K571. **(G)** The COM distance of F424-K571. **(H)** The COM distance of K351-E606. **(I)** The COM distance of K351-D602. **(J)** The COM distance of H336-Y609. **(K)** The COM distance of H340-Y609. **(L)** The COM distance of R311-E664.

A

Model	DOPE score	VERIFY 3D
1	-124585.95312	93.04%
2	-124642.69531	92.84%
3	-123915.72656	93.55%
4	-124408.96875	93.65%
5	-124679.29688	92.53%

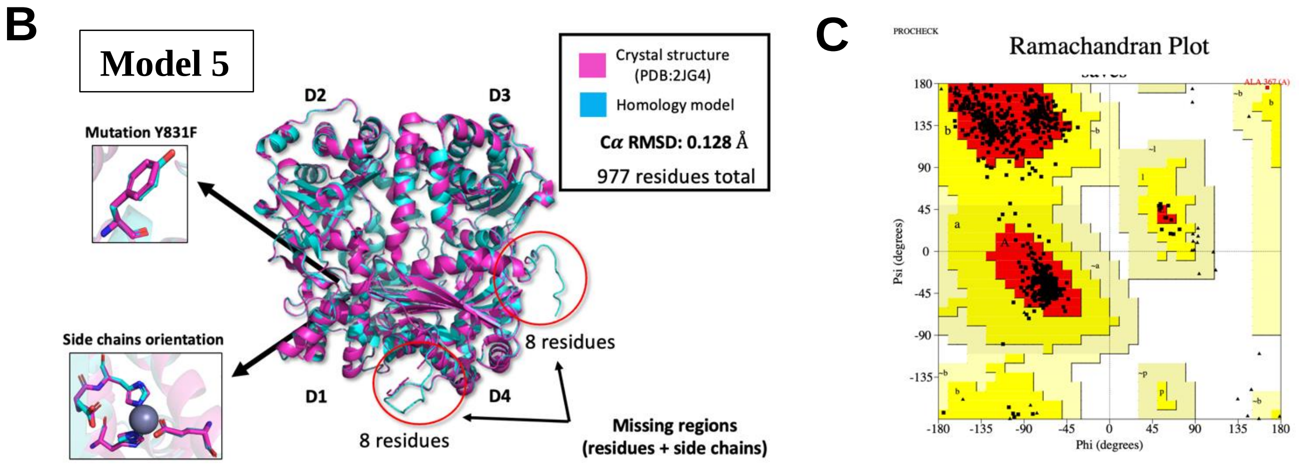

Figure S11. Model evaluation and selection after MODELLER analysis and generation. **(A)** Representation of the DOPE score and VERIFY 3D quality score for each model. **(B)** Details about missing residues and side chains, as well as the mutations fixed during the MODELLER analysis. Cα RMSD between the generated model (model 5) and the crystal structure (PDB:2JG4) has been calculated for further inspection. (**C**) Ramachandran plot of the selected model.
